# ESR Essentials: renal imaging in children—practice recommendations by the European Society of Paediatric Radiology

**DOI:** 10.1007/s00330-025-12100-3

**Published:** 2025-12-05

**Authors:** Magdalena Maria Woźniak, Damjana Ključevšek, Maria Beatrice Damasio, Luisa Lobo, Hans-Joachim Mentzel, Lil-Sofie Ording-Müller, Philippe Petit, Michael Riccabona, Samuel Stafrace, Anne M. Smets, Carmelo Sofia, Giulia Perucca

**Affiliations:** 1 Department of Paediatric Radiology Medical University of Lublin, Lublin, Poland; 2 Paediatric Radiology Unit, University Children’s Hospital, University Medical Centre Ljubljana, Ljubljana, Slovenia; 3 Department of Radiology, IRCCS Giannina Gaslini Institute, Genova, Italy; 4 Serviço de Imagiologia Geral, University Hospital, ULS Santa Maria, Lisbon, Portugal; 5 Section of Paediatric Radiology, Department of Radiology, University Hospital Jena, Jena, Germany; 6 Division of Radiology and Nuclear Medicine, Department of Paediatric Radiology, Oslo, Norway; 7 Hopital Timone-Enfant, Aix-Marseille University, Marseille, France; 8 Paediatric Radiology, Medical University Graz, Graz, Austria; 9 McMaster Children’s Hospital, Ontario, Canada; 10 Department of Radiology and Nuclear Medicine, Amsterdam UMC location University of Amsterdam, Amsterdam, The Netherlands; 11 Department of Biomedical Sciences and Morphologic and Functional Imaging, University of Messina, Messina, Italy; 12 Radiology Department, Great Ormond Street Hospital for Children NHS Foundation Trust, London, UK

**Keywords:** Kidney, Child, Diagnostic imaging, Magnetic resonance imaging, Workflow

## Abstract

**Abstract:**

Renal pathology is common in childhood. Imaging plays a critical role in the diagnosis of kidney diseases and encompasses a range of modalities. Advanced imaging is typically performed in specialised paediatric hospitals, where experienced paediatric radiologists are familiar with the relevant techniques, protocols, indications, and limitations. However, children are often first admitted to general hospitals, where radiologists may have more limited experience in paediatric imaging. Renal cysts in children differ from those in adults, most commonly presenting as cystic kidney diseases. In the majority of cases, ultrasound (US) is the sole diagnostic modality required. Imaging is not necessary for the diagnosis of urinary tract infection (UTI), but it is essential for detecting underlying anomalies and potential complications. Urinary tract dilatation is a common finding in children; however, only up to 30% of cases require further evaluation to diagnose urinary tract obstruction or vesicourinary reflux. Urolithiasis is relatively uncommon in children and is primarily diagnosed with US, although computed tomography (CT) may occasionally be necessary. Solid renal lesions identified on US should be further evaluated in highly specialised paediatric centres. Mild to moderate renal trauma can be diagnosed and monitored using US, whereas CT remains the modality of choice for assessing severe trauma.

**Clinical relevance statement:**

This review provides general radiologists with a comprehensive overview of the normal renal appearance across paediatric age groups, including normal variants and imaging pathways for the most common renal pathologies in children. It also highlights scenarios where referral to specialised paediatric centres is necessary.

**Key Points:**

- *US is the first-line imaging modality for diagnosing most renal pathologies in children*.
- *MRI is used in a variety of situations (e.g. complex congenital anomalies of the kidney and urinary tract, or focal lesions) when US is not sufficient*.
- *CT is reserved for emergencies and in selected cases of urolithiasis*.
- *Knowledge of normal renal features, including normal anatomical and morphological variants across different age groups, is essential*.

## Key recommendations

1. Ultrasound (US) is the modality of choice for evaluating renal pathologies such as cystic kidney lesions, urinary tract dilatation and other congenital anomalies, complications of urinary tract infections, renal calculi, mild and moderate renal trauma, and focal solid renal lesions (level of evidence: high).
2. Magnetic resonance imaging (MRI) is considered a radiation-free, comprehensive imaging technique for situations requiring detailed anatomical and morphological assessment (Level of evidence: high*)*. When functional MRI is applied, it can also provide functional information (level of evidence: moderate).
3. Computed tomography (CT) is a less preferred, second-line imaging modality in children, and should be limited to cases of major trauma or complicated urolithiasis considered on a case-by-case basis (level of evidence: high).

## Introduction

Renal pathologies are common in children, and imaging plays a vital role in both diagnosis and follow-up. The most frequent indications for renal imaging include assessment and monitoring of congenital conditions (e.g. collecting system dilatation, cystic renal disease), as well as acquired disorders such as urinary tract infection (UTI), urolithiasis, renal insufficiency, trauma and solid renal masses.

US is typically the first-line modality for diagnosis. Occasionally, MRI is indicated—for example, in evaluating suspected tumours, complex congenital anomalies, or unclear UTI complications. In rare cases, CT may be warranted, such as in severe trauma or complex stone disease [1]. Additional modalities, such as functional MR-Urography and scintigraphy, also play significant roles in the functional assessment of the kidneys.

This article provides guidance for general radiologists on how to approach common paediatric renal diseases. Rare or complex disorders are beyond the scope of this article, as such cases should be referred to specialised paediatric centres. Additional reading is available in radiology textbooks [2, 3].

### Imaging methods and normal variants

An overview of imaging techniques applicable in paediatric renal assessment is provided in Table 1, and common normal variants are outlined in Table 2. Regardless of the imaging modality, child-specific factors must be considered, including renal maturation, renal function, and age-specific renal appearance—especially in neonates and infants. Hydration status, bladder filling, posture and patient positioning all influence renal imaging and must be considered to distinguish normal variants from pathological findings (Table 2).

**Table 1:** Imaging methods applicable to childhood renal imaging—an overview

Method	Indication	How	Comments
General common methods			Experience in paediatric imaging is helpful
Abdominal radiography	Stone disease	Age-adapted dose, cone to region, including symphysis	Consider if it is really necessary
US	UTI, congenital anomalies, malformations and syndromes (CAKUT), including PCD, trauma, solid lesions, stones, hypertension and systemic disease, haematuria. nephrotic/nephritic syndrome, renal failure/insufficiency, cysts, dysplasia	Age-adapted transducers/frequencies (highest possible and applicable frequency); access ventrally/laterally and/or dorsally	In neonates/infants, use (phased) preferably linear (or micro-curved) high frequency transducers, do scans with good hydration and include bladder/ureters and adrenal gland; Colour/power and spectral Doppler if necessary
Advanced methods	Assure proper paediatric settings and experience	Usually done in dedicated paediatric radiology units	Except for CT in severe trauma (and some stones)
CEUS	ce-VUS—VUR assessment i.v. CEUS—focal renal lesion (complex cysts, UTI complication, tumour), and renal perfusion (APN, CPN, trauma)		i.v. CEUS is off-label, but renal-friendly—not excreted via the kidneys
MRI/MRU	Renal masses (tumour diagnosis, DDx/assessment and staging), complicated UTI, urinoma, complicated/complex severe PCD (e.g. PUJO), renal (split) function, perfusion, scars, urinary drainage—obstructive vs non-obstructive PCD.	T2 and (fs, ce-) T1 WS, rarely in-/out of phase and other sequences; DWI MRI, MR-angiography (e.g in renal hypertension); sedation in young children, feed and wrap in babies	Consider if Gd is necessary at all, or the T2-weighted water-MRU is sufficient, DWI suggested for inflammation and tumour
fMRU	Complex congenital anomalies of the kidney and urinary tract pre- and postoperative, are often considered the gold standard and radiation-free one-stop-shop imaging	T2 morphological, T2 water urographic and functional MRU with diuretic stress	Functional analysis on dynamic GD post diuretic sequence—including quantitative analysis
CT	Severe trauma, MRI not available (e.g. tumour assessment, complication in UTI unclear on US) or less helpful (e.g. complicated stone disease)	Use age/size/weight-adapted protocols, consider split bolus techniques if CA is necessary, and use weight-adapted CA dose	Avoid—except for the given indications. Sedation in young children; avoid multiphase scans
Scintigraphy	Renal (split) function, perfusion, scars, urinary drainage—obstructive vs non-obstructive PCD	Tc ^99m^ DMSA—static scan Tc ^99m^ MAG3—dynamic diuretic renography	Consider clinical impact, assure hydration for functional and diuretic scans

Methods that are used in the urinary tract, such as ce-VUS or fluoroscopy (VCUG), are not addressed, as they do not evaluate intrinsic renal disease—although they may be associated with or cause renal impairment

CEUS (advanced imaging, suggested for work-up in trauma, tumours or complicated cysts as an off-label application) will mostly be performed in dedicated centres and, as such, is not included in more detail in this table, which is aimed at advising general radiologists

*APN* acute pyelonephritis, *CA* contrast agent, *CAKUT* congenital anomalies of the kidney and urinary tract, *CEUS* contrast-enhanced ultrasound, *ce-VUS* contrast-enhanced voiding urosonography, *CPN* chronic pyelonephritis, *DDx* differential diagnosis, *DMSA* dimercaptosuccinic acid, *(f)MRU* (functional) magnetic resonance urography, *Gd* Gadolinium, *MAG3* mercaptoacetyltriglycin 3, *MRU* magnetic resonance urography, *PCD* pelvicalyceal dilatation, *PUJO* pelvi-ureteric junction obstruction, *Tc*^*99m*^ technetium, *UTI* urinary tract infection, *US* ultrasound, *VCUG* voiding cysto-urethrography

**Table 2:** Normal findings and variants in paediatric renal imaging, focused on US appearance

Diagnosis	Imaging method	Description	Example
Age-related changes			
Neonate	Mostly, only the US performed MRI, no indications for CT Functional studies are not recommended due to renal immaturity	Foetal lobulation; physiological echogenic cortex with marked cortico-medullary differentiation, rather spherical, mild physiological dilatation. Physiological medullary echogenicities should not be confused with nephrocalcinosis	
Infant	Mostly, only the US is performed, possibly using dorsal access, other modalities are used in well-defined indications	Less spherical, iso- or hypoechoic to liver parenchyma. No papillary echogenicities can be seen in neonates	
Child	US—mainstay of imaging, other modalities used in well-defined indications	Increasingly adult-like appearance: longer with ellipsoid shape, echogenic renal sinus, less pronounced cortico-medullary differentiation	
Normal and variants	Seen on all methods, the kidney parenchyma and shape are otherwise normal		
	**Definition**	**Comment**	**Image example**
Calyx and papilla—all ages	Sharp foramina and a rounded papilla are visible. A compound papilla is visible and normal if not associated with intrarenal reflux and scarring	With high resolution high frequency transducers visible in well-hydrated children—does not imply pathologic dilatation	
Renculation or persistent foetal lobulation	Physiologic indentation between units: each renal unit consists of a central medulla with a calyx and respective outer cortex	Should not be confused with a scar, which would point at the (clubbed) calyx with a thinned and altered cortical layer	
Parenchymal junction line/zone	The physiological border between the upper third and the rest of the kidney	Do not confuse with infection, duplication or scar	
Dromedary hump	A physiological contour bulge on the lateral border of the left kidney	Do not confuse with infection or tumour	
Column of Bertin	When 2 lobules fuse and deeper portions are resorbed, leading to a mass-like column of normal cortex (+ +)	May be small (left side) or hypertrophic (right side)—then do not confuse with tumour or infection, or duplex kidney. There may be a pyramid inside.	
Congenital anomalies of Kidney and Urinary Tract (CAKUT)—can still be variants without clinical sequelae or renal impairment			
Duplex kidney	Two separate pelvi-caliceal systems in one kidney, either with completely separate ureters or a split ureter/pelvis, can be recognised if the collecting systems show different dilatation	The two moieties are divided by a parenchymal bridge; they may be associated with reflux (lower system) or obstruction (upper moiety—Meyer Weigert rule). Ureters may insert ectopically—then not a normal variant	
Ectopic kidney	Normal kidney in abnormal position anywhere else in the abdomen (or even chest) may be smaller and often malrotated, shape may be somewhat distorted	Search for it also para-/retro-/supra-vesically if no kidney is found in the normal renal fossa, sometimes associated with congenital malformation of the ipsilateral genitalia (search for it)	
Horseshoe kidney	The two kidneys are fused anterior to the aorta in midline, the renal axis converges caudally, often malrotated	Normal if not associated with dilatation or dysplasia, often one side is smaller, higher risk of injury in trauma	

*CAKUT* congenital anomalies of the kidney and urinary tract, *CT* computed tomography, *MRI* magnetic resonance imaging, *US* ultrasound

US is the primary, and often only, modality used for the detection and follow-up of renal conditions. A thorough US evaluation should include the following:

- Kidney morphology and size (referencing standard values).
- Cortical echogenicity and corticomedullary differentiation (preserved, absent, or inverted).
- Evaluation of the renal hilum and collecting system (normal or dilated).
- Documentation of any existing pathological finding.

### Renal imaging in the paediatric cystic kidney disease

Cysts in an otherwise normal kidney are far less common in children compared to adults. A ‘simple renal cyst’ is rather an exception in children and may represent the manifestation of an underlying cystic kidney disease. Therefore, a child with an incidentally detected cyst should undergo a family history assessment and follow-up imaging. Localisation of the cyst (cortex/medulla) is important, as cortical cysts are generally less severe and less suggestive of an underlying disease [4, 5].

There is currently no universally accepted classification system for cystic kidney disease. It can be categorised based on the timing of development in relation to the stage of nephrogenesis, as hereditary (then further classified by genetic subtype) or as non-hereditary. Additionally, it is important to consider acquired cysts (e.g. post-traumatic) (Table 3). US imaging, which may initially show nonspecific or inconclusive findings, can reveal more characteristic patterns as the disease progresses. It is important to distinguish renal cysts from other entities such as calyceal dilatation or clubbing (which demonstrates a connection to the collecting system), a dilated upper pole system in a duplex kidney (characterised by an anechoic area surrounded by thinned parenchyma in the upper part), urinomas, or adrenal cystic changes (seen as anechoic cystic collections in the perirenal space).

**Table 3:** Paediatric cystic kidney disease—common* entities to consider, typical manifestations and appearance, and follow-up needs (table adapted from Gimpel et al [9], also using the chart from Riccabona et al [2, 3, 6, 38])

Entities	Manifestation	Follow-up or other imaging	Appearance
Non-genetic entities	May still be congenital, or manifest later in life (‘acquired cyst’)		
Cystic dysplasia/MCDK	Usually congenital, secondary to ureteric obstruction. Often associated with (ipsilateral) genital malformation—actively search for it. Cystic dysplasia can be bilateral. MCDK—no renal parenchyma, unilateral or bilateral form is lethal	US is used for monitoring the healthy kidney growth. Spontaneous resolution of MCDK	
Simple cyst	Often incidentally	Detailed medical and family history, thorough clinical examination, follow-up US—no CEUS, MRI or CT	
Acquired CKD	Usually depicted in the US after an event, e.g. after trauma	No follow-up, except for annual US in children after renal transplant and replacement therapy/chronic renal insufficiency, DDx caliceal diverticulum—contrast-enhanced magnetic resonance urography	
Complicated cyst	US, possibly CEUS (off-label use)	Contrast-enhanced MRI, if US (including CEUS) is unable to classify, apply the US or MRI-adapted ‘Bosniak’ classification	
Cystic tumour	Often palpable, sometimes as part of screening in tumour risk syndromes, e.g. haemorrhagic Wilms tumour, (segmental) cystic nephroma	CEUS + cross-sectional CE imaging (preferably MRI) for diagnostic work-up, staging and follow-up (unless benign—then US will suffice)	
Genetic CDK^*1^	Inherited—however may only manifest later in life, without congenital changes		
ARPKD	US (already prenatally), typically micro-cysts in enlarged kidneys, typical ciliopathy	Renal and abdominal US (liver cysts or fibrosis, dilated bile ducts, signs of portal hypertension); follow-up annually	
ADPKD I and II	Familial or imaging suspicion, vessel and liver/pancreas/spleen involvement common, typical ciliopathy	Confirm by US, follow-up by serial US, MRI if US restricted, suspicion of malignancy. Look for cysts in the liver, spleen and pancreas. In older patient, check for vessel complications (e.g. cerebral aneurysm)	

*The table presenting common and rare cystic lesions in children is included in the supplementary materials (Table S1).

*ADPKD* autosomal dominant polycystic kidney disease, *ARPKD* autosomal recessive polycystic kidney disease, *Ce* contrast enhanced, *CKD* chronic kidney disease, *MRI* magnetic resonance imaging, *DDx* differential diagnosis, *MCDK* multicystic dysplastic kidney, *MRU* magnetic resonance urography, *MRI* magnetic resonance imaging, *US* ultrasound

US description of cysts should include their appearance (simple or complex), number (solitary or multiple), laterality (unilateral or bilateral), location (cortical, medullar, corticomedullary junction, subcapsular or ubiquitous), distribution (diffuse or segmental), size (maximum diameter of the largest cyst), and internal characteristics (e.g. calcifications, wall thickening, septations, echogenic or floating content).

In complex cases, additional imaging may be required depending on the clinical question, or to add to in differential diagnosis (e.g. to differentiate a cystic tumour or a calyceal diverticulum). This may include CEUS or MRI.

Recommendations for evaluating cystic lesions in children have been published by several societies, including the European Society of Paediatric Radiology (ESPR) Abdominal Imaging Taskforce [6], the European Society of Paediatric Nephrology (ESPN) [7], and the Network for Early Onset Cystic Kidney Disease (NEOCYST) [8]. Procedural guidance can also be found in the international multidisciplinary consensus statement by Gimpel et al [9]. Assessment of extra-renal manifestations of cystic kidney disease is also recommended as part of the initial diagnostic workup if a diagnosis has not yet been established (Table 3).

In summary, renal cysts in children are most often manifestations of cystic kidney disease, which contrasts with their occurrence in adults. Imaging in suspected cystic kidney disease relies primarily on US. Complex cysts require further evaluation.

### Imaging in UTI

UTI is common in children. The history and clinical signs—particularly in younger children—are often non-specific. Diagnosis relies on urine analysis, including a positive urine culture. Imaging is needed to identify any anatomical and morphological abnormalities. Bladder dysfunction or congenital anomalies of the kidney and urinary tract (CAKUT), such as vesicoureteral reflux (VUR) or ectopic ureters, are risk factors for recurrent UTIs. Imaging is also essential in therapy-resistant UTIs to detect obstructions and to assess complications (Table 4).

**Table 4:** Possible manifestations of UTI in children as depicted by US

Diagnosis	US findings	Comment	Example
Acute pyelonephritis			
Renal size (length or volume)	Normal enlarged	Use nomograms for renal length due to considerable age/height variations	
Renal parenchyma structural changes	Loss of corticomedullary differentiation. Diffuse or focal altered echogenicity of the kidney (an-, hypo-, or hyper-echoic), renal sinus hyperechogenicity, decreased parenchymal perfusion in the affected areas, and subcapsular fluid in severe infections	Always assess areas of hypoperfusion with power Doppler and advanced small vessel imaging techniques, or occasionally i.v. CEUS (off-label)	
Urinary tract	Thickened urothelium with or without dilatation, Echogenic urine or debris in the collecting system	Hyperechogenic debris in urine is not specific for infection and must be correlated clinically	
Perinephric	Hyperechoic perinephric fat, pockets of fluid		
UTI complications			
Pyonephros	The fluid within the dilated pyelocalyceal system contains gravity-dependent, echogenic debris	The fluid may have few internal echoes and may suggest an uncomplicated hydronephrosis	
Abscess	Initially hypoechoic, hypoperfused area, Hypo/hyperechoic demarcated area, abscess capsule	CEUS (off-label use) is helpful to differentiate focal nephritis, abscesses	
Predisposing conditions			
Congenital anomalies of the kidney and urinary tract (CAKUT)	Obstruction of the urinary tract at different levels (PUJO, VUJO, LUTO), duplex kidney	Increased risk for VUR (evaluation with ce-VUS)	
Urinary stones	Present at different levels of the urinary tract	Predisposing factor for infection and obstruction Twinkling sign in differentiation of calcification	

*CAKUT* congenital anomalies of the kidney and urinary tract, *CEUS* contrast-enhanced ultrasonography, *ce-VUS* contrast-enhanced voiding urosonography, *LUTO* lower urinary tract obstruction, *PUJO* pelviureteric junction obstruction, *VUJO* vesicoureteric junction obstruction, *VUR* vesicoureteral reflux

There are varying recommendations in the literature regarding the indications for imaging in children with UTI. The decision to perform imaging during or after a UTI episode depends on the child’s age (younger children are more likely to undergo imaging due to a higher incidence of underlying CAKUT) and the clinical presentation (imaging is indicated in the upper UTI but not usually required in lower UTI, such as cystitis) [10–13].

US is the method of choice for assessing renal parenchymal abnormalities in febrile UTIs [14, 15]. There is no indication for imaging in isolated cystitis. Baseline sonography should include grey-scale imaging of the renal parenchyma and the surrounding tissues, along with volume estimation of each kidney. Colour Doppler should be used to detect hypoperfused areas relative to normally perfused renal parenchyma (Table 4). In complications and in the case of therapy failure, off-label use of contrast-enhanced ultrasound (CEUS) offers improved detection of focal nephritis, hypoperfusion, and abscess formation [16]. ^99m^T-dimercaptosuccinic acid (DMSA) scans are a traditional examination to evaluate renal parenchymal changes. DMSA can be replaced by MRI, including diffusion-weighted imaging (DWI), or CEUS [17, 18]. Acquired parenchymal lesions (e.g. chronic pyelonephritis) are risk factors for proteinuria, the development of hypertension, and end-stage renal disease (Table 4).

In summary, US is a valuable imaging tool for evaluating underlying pathology and complications in children with febrile UTI.

### Renal imaging in the paediatric pelvicalyceal dilatation (PCD)

PCD is one of the most common indications for US examination in early childhood, frequently identified initially on prenatal US. While the majority of cases are of no clinical significance and undergo spontaneous postnatal resolution, up to 30% of patients with prenatal PCD may develop UTI. Bilateral PCD can also be a sign of lower urinary tract obstruction (LUTO).

The principal aim of postnatal imaging is to assess the significance of the prenatal findings. In order to avoid underestimating the degree of dilatation, US should not be performed before the third postnatal day.

**Fig. 1:**
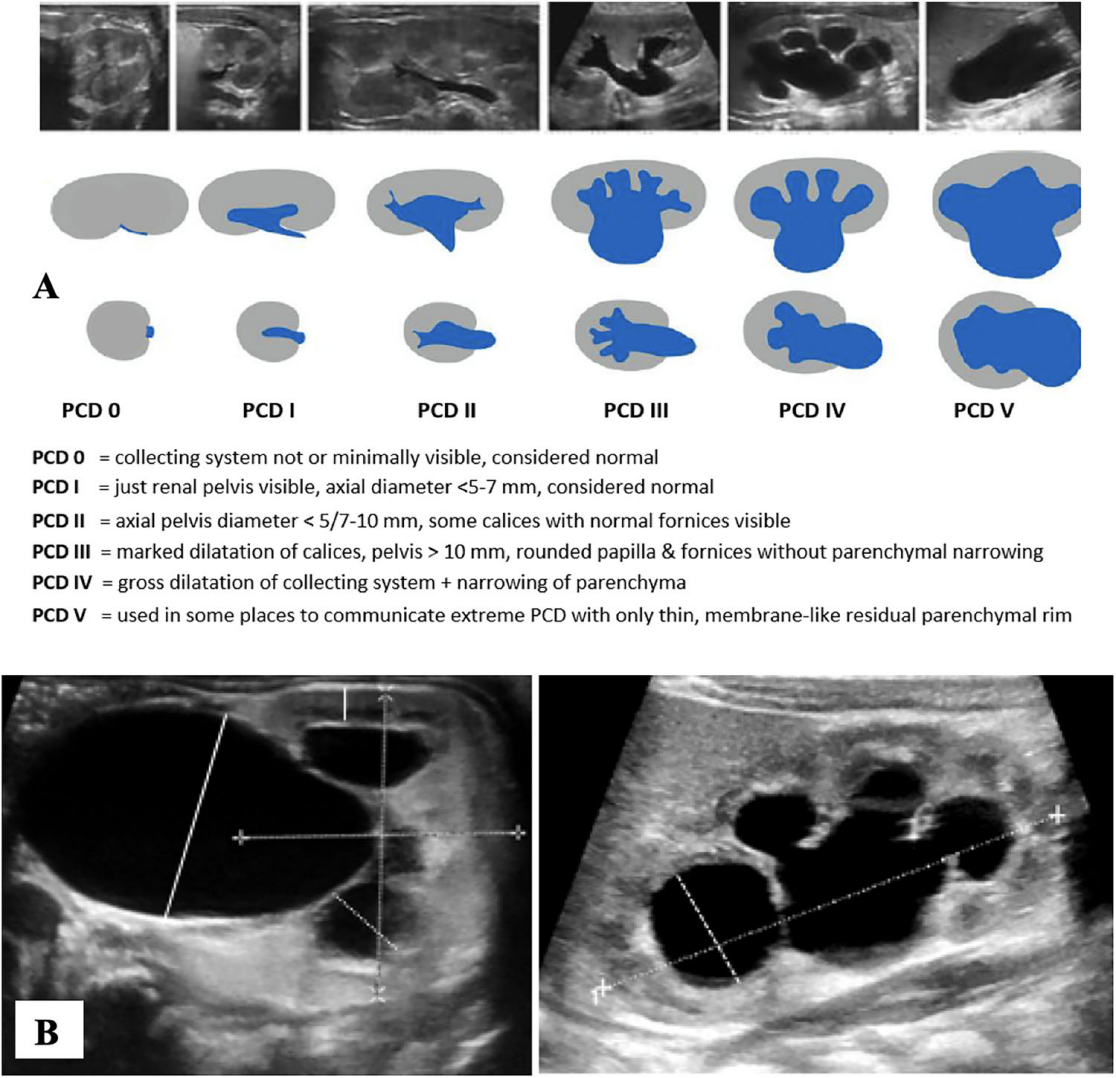
**A** PCD grading according to the ESPR abdominal imaging task force recommendation [20]. Descriptive approach focusing on caliceal configuration and renal parenchyma—measurements of minor importance; sufficient hydration mandatory. Ureter and bladder finings not included—however, these should be evaluated too (diameter, size, wall, ureteric peristalsis, residual volume, bladder neck. NOTE: A visible collecting system does NOT imply pathology. **B** How to properly measure PCD (calices, intra- and extrarenal pelvis, parenchymal thickness, renal diameters). Perform all measurements in a standardised fashion—assure proper hydration and the same position as well as bladder filling for every examination (prone/supine)

US assessment should aim to:

1. Grade PCD by:
  Measuring the pelvicalyceal system (PCS) using standardised techniques (Fig. 1A, B).
  Describing the shape and degree of calyceal dilatation (which may require further evaluation).
  Assessing the renal parenchyma (cortical thickness, echogenicity, presence of dysplastic cysts).
  Estimating the renal size by calculating the total renal volume.
2. Assess the entire urinary tract, including the bladder, ureters, and urethra, as these findings are integral to interpreting the significance of PCD.
3. Identify the underlying condition, which may include:
  Pelvi-ureteric junction (PUJ) obstruction,
  Uretero-vesical junction (UVJ) obstruction,
  Caliectasis without obstruction,
  Dilating VUR,
  Signs suggestive of LUTO.

In many cases, additional imaging is necessary to establish a definitive diagnosis. Voiding cystourethrography (either contrast-enhanced US or radiographic voiding cystourethrography) is indicated where VUR or bladder outlet obstruction is suspected. Functional assessment and evaluation of obstruction severity are typically performed using Technetium (Tc^99m^) mercaptoacetyltriglycine (Tc^99m^ MAG3) scintigraphy or contrast-enhanced functional MR urography (fMRU). For evaluating complex urinary tract anatomy, T2-weighted “water MRU” can be employed. This technique has largely replaced intravenous urography [19–24].

Follow-up is generally recommended for dilatations of grade 3 or higher, although specific diagnostic algorithms may vary depending on local protocols and clinical presentation.

If bladder outlet obstruction is suspected or deterioration occurs during follow-up—or in cases of recurrent UTIs—patients should be referred to a specialist paediatric urology unit for further evaluation.

In summary, the US is the ideal first-line imaging modality for assessing the degree of PCD in childhood and is usually sufficient. However, in cases of suspected VUR or urinary tract obstruction, referral to a specialised unit is warranted for further imaging and potential surgical management.

### Urolithiasis

Urolithiasis refers to the accumulation of stones along the urinary tract, a condition that can be challenging to diagnose in children, particularly newborns and infants, due to its non-specific symptoms. Many children remain asymptomatic and are diagnosed incidentally during imaging examinations. Underlying metabolic abnormalities (such as hypercalcemia, hyperoxaluria, hypocitraturia, and cystinuria), UTIs, urinary tract malformations and diversions, low fluid intake, and high sodium intake are the most frequent risk factors for paediatric urolithiasis. Most renal stones in children are composed of calcium oxalate and calcium phosphate; struvite or cystine stones are less common.

**Fig. 2:**
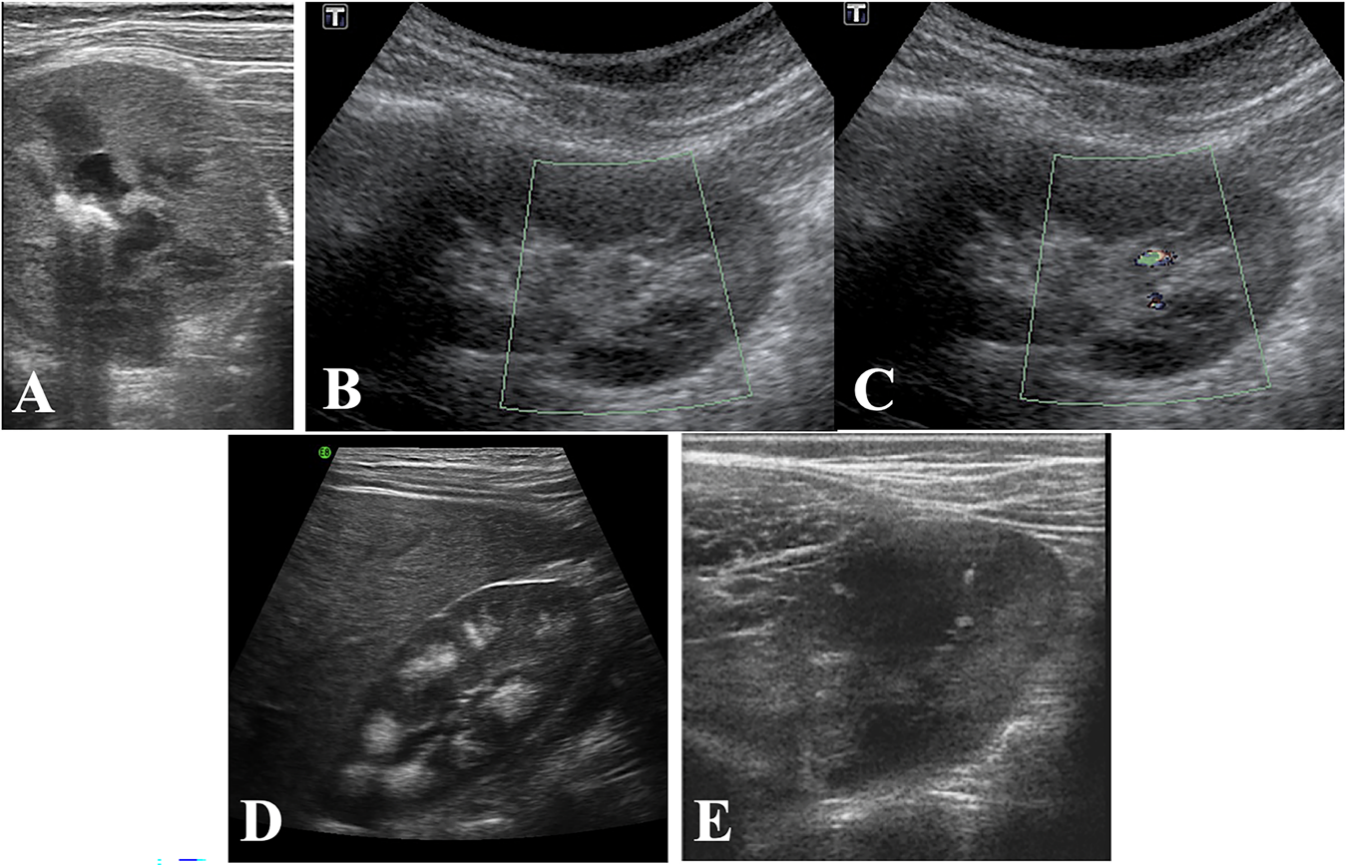
**A** Appearance of stone with a high US frequency probe: hyperechoic structure with posterior shadowing. **B** Sagittal section of the kidney with a low-frequency probe showing multiple hyperechoic foci due to interface with vessel walls. **C** The use of Doppler with high PRF reveals one of them to be small calculi without posterior shadowing. **D** Sagittal section of the kidney showing hyperechoic material centred on the pyramids without posterior shadowing corresponding to nephrocalcinosis. **E** Multiple hyperechoic foci in the cortex and the medulla corresponding to a tiny angiomyolipoma in this known patient, followed up for tuberous sclerosis

US is recommended as the initial diagnostic modality in children, where calculi appear as hyperechoic structures within the PCS, with or without posterior shadowing (Fig. 2A), depending on the stone’s size. The accuracy of US can be enhanced by using colour Doppler, which may produce the ‘twinkling artefact’, defined as the appearance of alternating colours behind a reflective object (Fig. 2B, C). Increasing the pulse repetition frequency (PRF) to suppress background colour signal and angling the US beam at various positions improves identification and visualisation of this artefact. The focal zone should be positioned below the stone; placing it at or above the stone may weaken the artefact. However, the twinkling artefact does not differentiate calcifications from stones.

Several conditions may mimic kidney stones on US. Medullary nephrocalcinosis (Fig. 2D), characterised by calcium deposits in medullary pyramids, can be distinguished from nephrolithiasis by the specific localisation of echogenic foci outside the collecting system and the absence of acoustic shadowing. In neonates, Tamm-Horsfall protein accumulation, which results in transient pyramidal echogenicity, must be considered. Angiomyolipomas (AMLs) (Fig. 2E), which appear hyperechoic like stones, can be differentiated by their intraparenchymal or exophytic localisation and lack of calcification. Air in the collecting system following catheterisation is another finding that may be misinterpreted as calculi.

Kidney, ureter, and bladder (KUB) radiography is no longer used, as most stones are too small or insufficiently calcified to be visible, and it has been largely replaced by ultra-low-dose non-contrast CT protocols, which offer high accuracy with minimal radiation exposure. Ultra-low-dose CT is defined as delivering a radiation dose lower than 50% of the expected normal dose for CT and less than 30% of that of low-dose CT [25, 26]. The reported mean effective radiation dose of optimised ‘stone-protocol CT’ ranges from 0.8 to 2.5 mSv, compared to approximately 0.01–0.11 mSv of KUB radiography [27]. Nonetheless, CT should only be performed when further imaging is required to guide management and when US is inconclusive.

MR urography (MRU) provides detailed anatomical and functional information but rarely visualises stones, which appear as signal voids and can therefore be missed. Consequently, MRU is not recommended for stone detection. [28].

In summary, US is an accurate method for stone detection, demonstrating hyperechoic foci and the twinkling sign. CT is reserved for inconclusive or complex cases.

### Imaging in paediatric solid renal masses

**Fig. 3:**
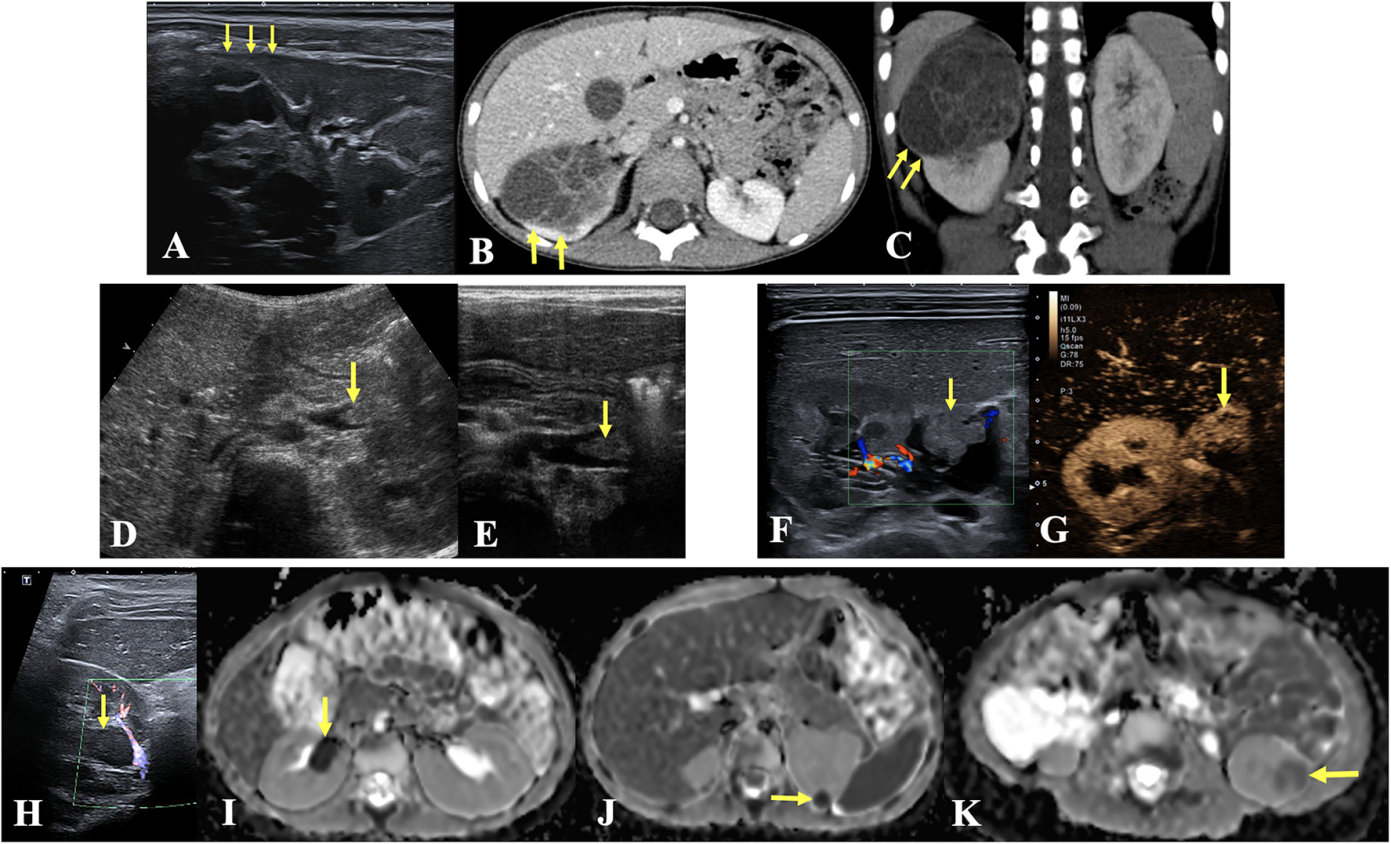
**A–C** Four-year-old boy involved in a car accident. Incidental finding on a CT scan done in a trauma setting. US and repeat CT days later clearly demonstrate (yellow arrows) a smooth angle between a cystic tumour and the adjacent normal parenchyma. This claw sign indicates the renal origin of this tumour, which was confirmed on pathology to be a cystic nephroma. **D**, **E** Four-year-old girl. US workup of a large left nephroblastoma revealed a tumour thrombus within the left renal vein (yellow arrows) (**D**—convex probe; **E**—linear probe). **F** Fourteen-month-old boy with a renal mass with a mixed echotexture on a horseshoe kidney. No flow was detectable on Colour Doppler within the echogenic portion of the mass. **G** Contrast-enhanced US shows the vascularised aspect of the lesion and rules out thrombus/debris. **H** Three-year-old girl with known Beckwith–Wiedemann syndrome. Screening US revealed a hypoechoic perihilar mass within the right kidney. **I**–**K** On apparent diffusion coefficient maps, the right renal lesion is easily depicted, as well as two other lesions not visible on US in the left upper and lower kidney, in keeping with nephroblastomatosis vs Wilms tumour

The discovery of solid renal masses in children is most often made clinically, due to their typically large size, or during a follow-up in a high-risk population (e.g. Beckwith–Wiedemann syndrome, tuberous sclerosis). Less commonly, these masses are detected post-traumatically, incidentally, or on foetal US. Regardless of the child’s age, nephroblastoma is by far the most common renal tumour—except in neonates, where mesoblastic nephroma has a higher incidence. US examination using both low- and high-frequency probes, is the cornerstone of the initial assessment and must address the following key questions:

- Is the lesion truly intrarenal? This distinction can be subtle. Evaluating the angles between the renal parenchyma and the lesion is helpful (Fig. 3A–C). The position of the renal hilar vessels (displaced in renal tumours, encased in extrarenal tumours—mostly neuroblastoma) is also informative.
- Are there features suggestive of a benign tumour or an inflammatory lesion (e.g. lobar nephroma, AML, etc.; see Table 5)?
- Is the lesion solitary, or are there multiple lesions present? Is it unilateral or bilateral (e.g. bilateral nephroblastoma versus nephrogenic rests)?
- What are the lesions’ characteristics? Consider its size, margins, echotexture (solid, cystic, mixed, with or without calcifications), and vascularity.
- Is there involvement of the renal vascular pedicle? Look for signs of (compression or thrombosis in the renal vein, inferior vena cava and/or right atrium) (Fig. 3D, E), as well as any effect on the renal collecting system (e.g. distension or invasion).
- Is another abdominal or pelvic organ involved? This may aid in differential diagnosis (e.g. lymphoma) or indicate metastasis, which suggests a more aggressive renal tumour.

The next step involves planning further imaging and defining a management strategy based on the US findings. To avoid unnecessary repeat imaging, further evaluation (MRI or CT) and decisions regarding biopsy should be coordinated at a specialised centre.

**Table 5:** List of common* solid renal lesions in childhood

Entity	Age group	Description	Remark
Variations			
Hypertrophic column of Bertin	Any age	See table ‘Normal findings and variations’	DDx—hypertrophic parenchyma in scarring
Parenchymal bridge in a duplex system	Any age	On US: central echogenicity disrupted by normal renal parenchyma without mass effect, 2 renal pelvises	Possible accessory renal vessels
Dromedary hump	Any age	Lateral contour alteration in the mid-third of the kidney with normal parenchyma	Only the left kidney
Inflammatory			
Focal nephritis	Any age	On US: hypo- or hyperechoic ‘pseudo-mass’ confined to the medulla	Diagnosis made clinically and by follow-up, usually, no other imaging other than US is necessary
Inflammatory pseudotumour/lobar nephroma	Any age Rather rare	On US: hypoechoic homogeneous mass with sharp margins	Don’t start chemotherapy unless an inflammatory process is excluded
Abscess, necrosis, haemorrhage	Any age Rather rare	Varying echogenicity, no central vascularisation	If the US is clear, no other imaging is needed
Benign and intermediate malignant			
AML	Any age Very rare in patients without tuberous sclerosis	On US: mixed echogenicity, often rather hyperechoic, often multiple; echogenicity at least equal to fat in the hilum	May bleed, also seen in other abdominal parenchymal organs, particularly in the liver If little or no fat, consider epithelioid AML (prognosis different—need for biopsy)
Nephroblastomatosis/nephrogenic rests	Can be pre-malignant, > 2 years	Can easily be missed on US, CEUS may be helpful	MRI with DWI is recommended
Malignant			
Wilms tumour (WT)	Mean age 3 years (and older), the most common renal tumour in this age group, but can be present even before birth and in neonates	90% of paediatric renal tumours, usually solid, homogeneous masses, mostly exophytic, often with a pseudo-capsule; calcifications are rare	99% of WT tumours are treated without biopsy, based only on imaging findings—according SIOP protocol, biopsy is recommended only with atypical clinical, imaging or biological features

*AML* angiomyolipoma, *CEUS* contrast-enhanced ultrasound, *DDx* differential diagnosis, *DWI* diffusion-weighted imaging, *MRI* magnetic resonance imaging, *US* ultrasound, *SIOP* International Society of Paediatric Oncology, *WT* Wilms tumour

Warning: do not biopsy a renal mass; refer affected children to a specialised centre for further imaging, staging, further workup, and treatment according to the European study protocols

*The table presenting common and rare solid lesions in children is included in the supplementary materials (Table S2)

In summary, the US can, in most cases, differentiate tumorous masses from inflammatory lesions. Suspected renal masses must be referred to specialised centres.

### Renal trauma in paediatrics—imaging modalities

**Fig. 4:**
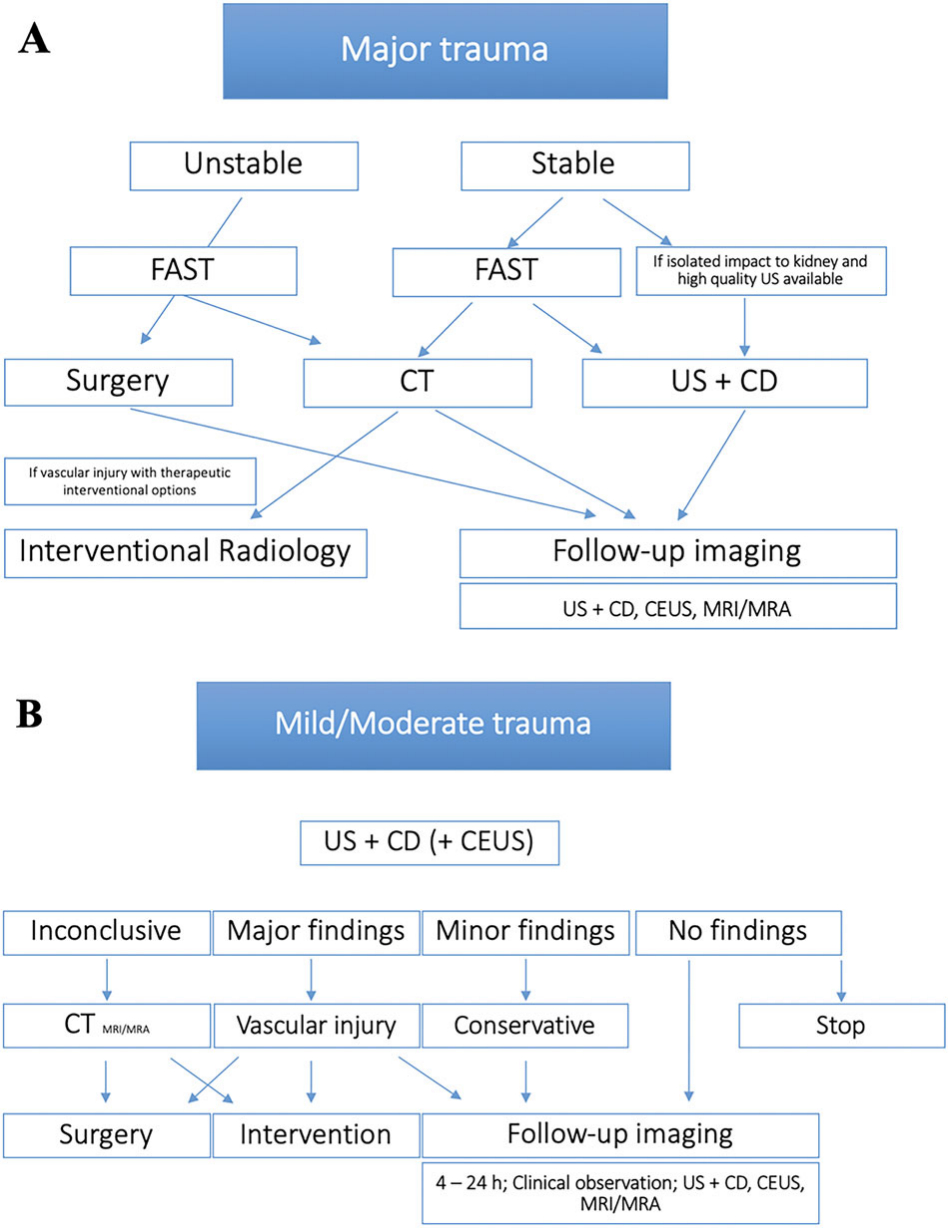
**A** Imaging algorithm in suspected severe renal trauma. Pre-test likelihood. E.g. rapid deceleration injury, direct flank injury with high impact, fall from height. **B** Imaging algorithm in mild to moderate renal trauma

Children are at increased risk of kidney injury from blunt abdominal trauma due to a relatively thin perirenal fat layer, weaker abdominal wall muscles, and more elastic ribs, compounded by the relatively larger renal size and increased kidney mobility (fixation occurs only via vascular pedicles and ureter) [29]. Consequently, approximately 5–20% of children sustaining blunt abdominal trauma will also have renal injury [30]. Injury grading is based on the American Association for the Surgery of Trauma (AAST) organ injury scale. A significant indicator of renal trauma is a history of rapid deceleration events (e.g. pedestrian, bicycle, or vehicle accidents, and falls from height) (Fig. 4A, B).

**Fig. 5:**
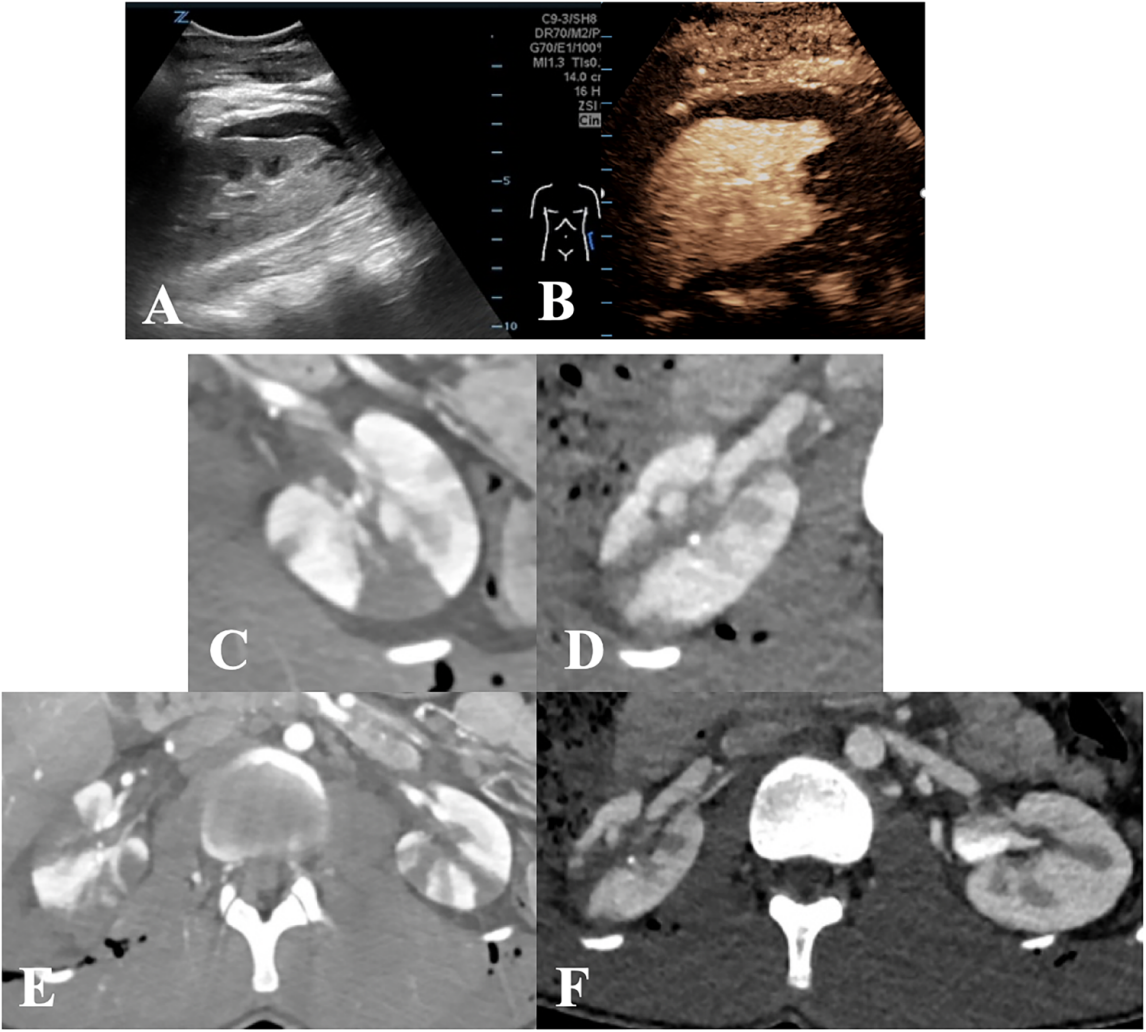
**A**, **B** Eight-year-old male, bicycle accident with a sport utility vehicle (SUV). **A** Laceration and fracture of the kidney (AAST III) and spleen (AAST IV). **B** Contrast-enhanced ultrasound (CEUS) in the intensive care unit because of instability for transport to the CT unit. 0.8 mL SonoVue applied. Conservative management without complications. **C**–**F** Seventeen-year-old male, motorcycle accident. Severe spinal trauma with paraplegia. Temporary vascular segmental lesions of both kidneys, laceration of the right kidney. No urinoma

The main objectives of imaging in renal trauma are to accurately stage the injury (including vascular supply, parenchymal lacerations, segmental infarcts, collecting system involvement, and urinary extravasation), identify pre-existing renal and urinary tract pathologies, assess the function of the affected and contralateral kidney, and detect associated injuries in other organs [31] (Fig. 5A–F).

The initial radiological assessment in blunt abdominal trauma is focused abdominal sonography for trauma (FAST), which has high specificity, variable sensitivity, and a low negative predictive value. The US with colour Doppler can diagnose and exclude clinically significant renal injuries [32]. However, limitations include an inability to distinguish extravasated urine from fresh blood. Colour and pulsed Doppler are necessary to identify vascular pedicle injuries. Visualisation may be compromised by open wounds, rib fractures, bowel dilatation from intestinal ileus, and obesity. Additionally, the technique is highly operator-dependent and can be time-consuming.

CT with intravenous contrast is the imaging modality of choice in moderate to severe abdominal trauma in children [33]. The split bolus contrast technique and excretory phase CT should be employed to avoid the ‘classic’ four-phase CT protocol, which includes non-contrast, arterial, nephrographic, and pyelographic phases. Intravenous contrast medium (iodine concentration of 300 mg/mL) is usually administered at a dose of 1.5–2 mL/kg body weight and an injection rate of 2–4 mL/s via a cubital vein.

Angiography may be required for embolisation of active bleeding or intervention in cases of renal artery dissection. MRI might be reserved for follow-up imaging.

Follow-up imaging is recommended at day 1 (4–24 h), and again at 7–10 days post-trauma to assess for secondary bleeding, arteriovenous fistulae, or developing pseudoaneurysms [34, 35]. Contrast-enhanced ultrasound (CEUS) is recommended by the European Federation of Societies for Ultrasound in Medicine and Biology (EFSUMB) for follow-up of conservatively managed abdominal trauma [36]. However, injuries of the collecting system cannot be evaluated with CEUS, as microbubbles are not excreted by the kidneys [37].

In summary, US is the first-line imaging modality for renal injury detection in blunt abdominal trauma, while CT is the method of choice in moderate and severe cases.

## Summary statement

Renal imaging in children is common in clinical practice. The radiologist plays a crucial role in evaluating suspected renal pathology, which differs from adult renal diseases. Paediatric renal pathology encompasses CAKUT, cystic kidney disease, UTIs and their complications, focal solid renal lesions, urolithiasis, and renal trauma.

The primary purpose of renal US as a first-line imaging modality is the early detection of pathological changes that may lead to chronic renal failure. Consequently, the radiologist must be familiar with the normal renal appearance across different age groups, common normal renal variants, and fundamental pathological alterations.

When the US is insufficient to answer the clinical question, MRI or CT, as well as functional renal imaging techniques (such as functional MRU and renal scintigraphy) are the next imaging options. These are typically performed by paediatric radiologists in non-urgent settings.

## Patient summary

Renal US is the method of choice in children with suspected renal pathology. It is non-invasive, widely available, relatively fast, dynamic, safe, and radiation-free. This article provides a summary of US findings for the most common renal pathologies in children, along with guidance on recognising conditions that require further evaluation with appropriate imaging techniques and timely management to prevent the progression to chronic kidney disease.

## Supplementary information

ELECTRONIC SUPPLEMENTARY MATERIAL

## Abbreviations

AAST: American Association for the Surgery of Trauma
AML: Angiomyolipoma
CAKUT: Congenital anomalies of the kidney and urinary tract
ce-VUS: Contrast-enhanced voiding urosonography
CEUS: Contrast-enhanced ultrasound
CT: Computed tomography
DDx: differential diagnosis
DMSA: 99mT-dimercaptosuccinic acid scan
DWI: Diffusion weighted MRI
KUB: Kidney, ureter, bladder
LUTO: Lower urinary tract obstruction
MAG3: Mercaptoacetyltriglycin 3
MRI: Magnetic resonance imaging
MRU: Magnetic resonance urography
PCD: Pelvicalyceal dilatation
PCS: Pelvicalyceal system
PRF: Pulse repetition frequency
Tc 99m: Technetium
Tc^99m^ MAG3: Dynamic diuretic renography
US: Ultrasound
UTI: Urinary tract infection
VUR: Vesicoureteral reflux
