# Supplementary material for: ESR Essentials: renal imaging in children—practice recommendations by the European Society of Paediatric Radiology
Source: Eur Radiol. 2025 Dec 5;36(5):3611–27. doi: 10.1007/s00330-025-12100-3 (PMC13086778; doi:10.1007/s00330-025-12100-3)
Supplement: Supplementary file 1 — ELECTRONIC SUPPLEMENTARY MATERIAL [file 330_2025_12100_MOESM1_ESM.pdf]

ESR Essentials: renal imaging in children - practice recommendations by the European Society of Paediatric Radiology

Electronic Supplementary Material

Table S1. Paediatric cystic kidney disease – common and rare entities to consider, typical manifestation and appearance, and follow-up needs (Table adapted from Gimpel C et al. [9], also using the chart from Riccabona M et al. [2; 3; 6; 10])

Entities	Manifestation	Follow-up or other imaging	Appearance
**Non-genetic entities**	May still be congenital, or manifest later in life (‘acquired cyst’)		
**Cystic dysplasia (can be bilateral) / MCDK(no renal parenchyma, unilateral as bilateral form is lethal)**	Usually congenital, secondary to ureteric obstruction<br>Often associated with (ipsilateral) genital malformation – actively search for it	Depends on if bilateral and if with or without contralateral renal hypertrophy, risk of progression –US used for follow-up though often with little contribution to the further course and for monitoring the healthy kidney growth if unilateral	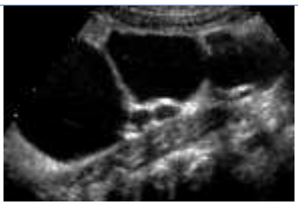
**Simple cyst**	Often incidentally	Detailed medical and family history, thorough clinical examination, follow-up US – no CEUS, MRI or CT	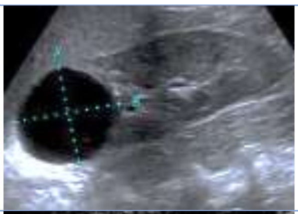
**Acquired CKD**	Usually depicted on US after event, e.g., after trauma	no follow-up, except for annual US in children after renal transplant and replacement therapy / chronic renal insufficiency<br>DDx caliceal diverticulum - ce-MRU	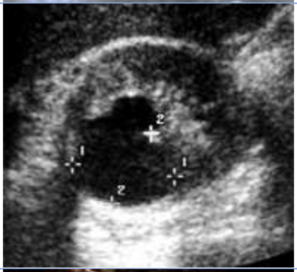
**Complicated cyst**	US, possibly CEUS (off label use)	Ce-MRI, if US (including CEUS) is unable to classify<br>Apply US-or MRI-adapted ‘Bosniak’ classification	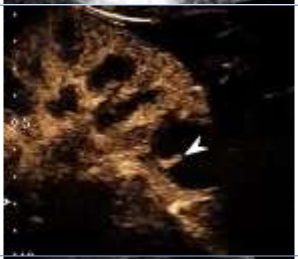
**Cystic tumour**	Often palpable, sometimes as part of screening in tumour risk syndromes<br>e.g. haemorrhagic Wilms tumour, (segmental) cystic nephroma …	CEUS + cross-sectional ce- imaging (preferably MRI) for diagnostic work-up, staging and follow-up (unless benign – then US will suffice)	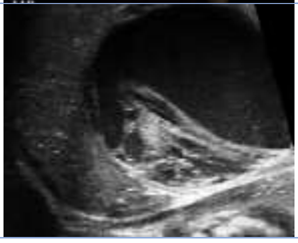
**Genetic CDK*1**	Inherited – however may only manifest later in life, without congenital changes		

**ARPKD**	US (already prenatally), typically micro-cysts in enlarged kidneys<br>Typical ciliopathy	Renal and abdominal US (liver cysts or fibrosis, dilated bile ducts, signs of portal hypertension); follow-up annually	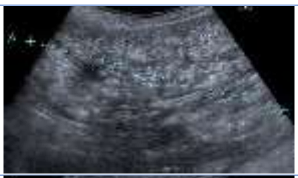
**ADPKD I and II**	Familial or imaging suspicion, vessel and liver/pancreas/spleen involvement common<br>Typical ciliopathy	Confirm by US, follow-up by serial US, MRI if US restricted, suspicion of malignancy<br>Look for cysts in liver, spleen and pancreas, in older patient check for vessel complications (e.g. cerebral aneurysm)	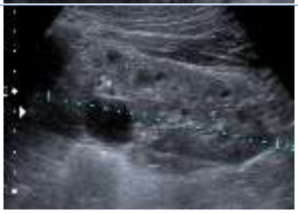
**Nephronophthisis**	Seen on US<br>Association GCKD, MCKD, and other CKDs<br>ciliopathy	No regular (US) follow-up<br>Look for liver involvement in conditions with known extra-renal manifestations<br>MCKD - medullary cysts<br>GCKD - dilated bowman capsule resulting in mostly subcapsular and cortical cysts	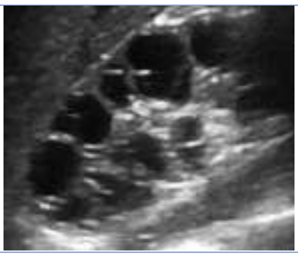
**ADTKD UMOD and MUC1 (former MCKD)**	Usually seen on US, includes also (segmental) 'medullary sponge kidney'<br>Uromodulin defect	Depends on size and clinical presentation, under discussion, at least US	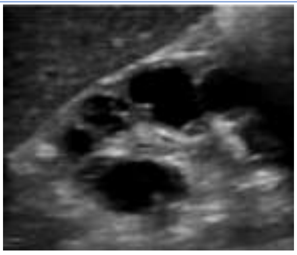
**ADTKD HNF1b**	On US kidneys may just be hypo- or dysplastic, do not need to exhibit depictable cysts<br>Uromodulin defect	Associated genital, pancreatic, hepatic and thymus anomalies - follow-up depending on structural kidney involvement<br>At least once evaluate genitalia, liver and pancreas (often with early onset diabetes)	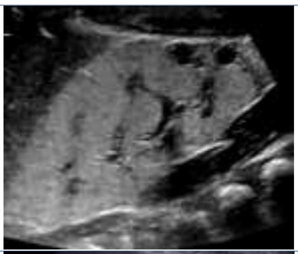
**TS-complex (classical and others)<br>BBS**	Seen on US, BBS consists of microcysts arising at the corticomedullary junction (dilated tubules)<br>ciliopathy	Regular annual US follow-up, possibly initial MRI and if unclear	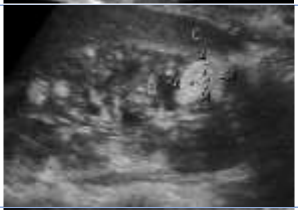
**Syndromic**	Joubert, Bardet-Biedl, Meckel-Gruber<br>Rare with Ivemark Sy, Hajdu-Cheney Sy, Zellweger Sy, Beckwith-Wiedemann Sy	Follow-up US imaging to assess progression of disease if of clinical impact	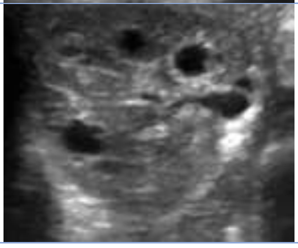

Note: (US) imaging appearance initially often unspecific and indecisive, may become more specific by typical patterns when disease evolves. Do not confuse caliceal dilatation or clubbing, a dilated upper pole system in a duplex kidney, an urinoma or adrenal cystic changes with renal cysts!

[*1] In ciliopathies (hereditary defect of cilia) heterogeneous phenotypes and many organs involved (as well as in syndromic cysts and other genetic disorders) – even thymus, genitalia central nervous system or musculo-skeletal system affected
Often genetic conditions manifest already initially bilaterally – particularly ARPKD

Abbreviations: ARPKD - autosomal recessive polycystic kidney disease, ADPKD - autosomal dominant polycystic kidney disease, ADTKD - tubulo-interstitial CKD, BBS - Bardet-Biedel Syndrome, CEUS - contrast-enhanced ultrasound, ce - contrast-enhanced, ESPR - European Society for Paediatric Radiology, GCKD - glomerulocystic kidney disease, MCDK - multicystic dysplastic kidney, MCKD - medullary cystic kidney disease, TS - tuberous sclerosis, US – ultrasound

Table S2. List of common and rare solid renal lesions in childhood

Entity	Age group	Description	Remark
**Variations**			
**Hypertrophic column of Bertin**	Any age	See table 'Normal findings and variations''	DDx - hypertrophic parenchyma in scaring
**Parenchymal bridge in a duplex system**	Any age	On US: Central echogenicity disrupted by normal renal parenchyma without mass effect, 2 renal pelvises	Possible accessory renal vessels
**Dromedary hump**	Any age	Lateral contour alteration in mid-third of kidney with normal parenchyma	Only left kidney
**Inflammatory**			
**Focal nephritis**	Any age	On US: hypo- or hyperechoic 'pseudo-mass' confined to the medulla	Diagnosis made clinically and by follow-up, usually no other imaging other than US necessary
**Inflammatory pseudotumour / lobar nephroma**	Any age<br>Rather rare	On US: hypoechoic homogeneous mass with sharp margins	Don't start chemotherapy unless an inflammatory process is excluded
**Xanthogranulomatous pyelonephritis**	Any age<br>Rare	Typically, inhomogeneous and destructive aspect with clubbed and dilated pelvi-caliceal system, staghorn stone / calcifications	Often additional CT or MRI indicated
**Abscess, necrosis, haemorrhage**	Any age<br>Rather rare	Varying echogenicity, no central vascularisation	If US is clear, no other imaging is needed
**Benign and intermediate malignant**			
**Mesoblastic nephroma**	Neonate / congenital (<8 months)<br>Rather rare	Solid homogenous or heterogenous mass; can invade the perinephric space<br>Hypercalcemia	'semi'-malignant, treatment - nephrectomy without further chemotherapy or staging / follow up (if histology confirms initial suspicion)
**Hamartoma**	Any age<br>Extremely rare	Variable appearance on US, isoechoic, may contain calcifications and fat	Often associated with syndromes
**Angiomyolipoma (AML)**	Any age<br>Very rare in patients without tuberous sclerosis	On US: mixed echogenicity, often rather hyperechoic, often multiple; echogenicity at least equal to fat in the hilum	May bleed, also seen in other abdominal parenchymal organs, particularly in the liver<br>If little or no fat consider epithelioid AML, (prognosis different - need for biopsy)
**Ossifying renal tumour of infancy**	Early infancy<br>Very rare	Gross calcification, little visible other tumour stroma, may compress collecting system	
**Nephroblastomatosis / nephrogenic rests**	Can be pre-malignant, > 2 years	Can easily be missed on US, CEUS may be helpful	MRI with DWI is recommended
**Cystic nephroma / multilocular cystic renal tumour**	Usually older (> 4 years) but can occur at any age<br>Relatively rare	Tumour containing numerous small cysts separated by thin septae; different from adult type	If thick and irregular septa or nodular component: caveat malignancy, indistinguishable from WT
**Malignant**			
**Wilms tumour (WT)**	Mean age 3 years (and older),	90% of paediatric renal tumours, solid usually homogeneous mass, mostly	99% of WT tumours are treated without biopsy based only on imaging findings –

	most common renal tumour in this age group, but can be present even before birth and in neonates	exophytic, often with a pseudo-capsule; calcifications are rare.	According SIOP protocol biopsy recommended only with atypical clinical, imaging or biological features
**Rhabdoid tumour**	Young infants, mean age 18 months<br>Relatively rare	Often subcapsular fluid collections, no pseudo-capsule, ill-defined margins, calcifications, hypercalcemia, early systemic metastasis (lung, bone, brain),	Highly aggressive, poor prognosis<br>Note: synchronous or metachronous intracranial tumour
**Clear cell sarcoma**	Mean age 3 years, > 6 months<br>Rare	Often partially cystic.<br>Bone metastasis	Quite aggressive
**Lymphoma (NHL)/ leukaemia (mostly AML)**	> 3 / 5 years<br>Relatively rare	Focal hypoechoic mass or diffuse renal enlargement by infiltration	Intermediate prognosis
**Juvenile renal cell carcinoma**	> 10 years<br>Relatively rare, though more common than Wilms tumour in this age group	Same features as in adults	Intermediate prognosis

Abbreviations: AML – angiomyolipoma, CEUS – contrast-enhanced ultrasound, CT – computed tomography, DDx – differential diagnosis, DWI – diffusion-weighted imaging, MRI – magnetic resonance imaging, NHL – non-Hodgkin lymphoma, US – ultrasound, SIOP - International Society of Paediatric Oncology, WT – Wilms tumour.

Warning: Do not biopsy a renal mass; Refer affected children to a specialised centre for further imaging, staging, further workup, and treatment according to the European study protocols
